# Addressing Vulnerable Population Needs in the Last Mile to the elimination of mother to child transmission of HIV: (Re)Claiming the HIV Response for Female Sex Workers and Their Children

**DOI:** 10.1186/s12889-020-09114-5

**Published:** 2020-06-26

**Authors:** Avi J. Hakim, Tegan Callahan, Irene Benech, Monita Patel, Michelle Adler, Surbhi Modi, Moses Bateganya, Kae Anne Parris, Trista Bingham

**Affiliations:** US Centers for Disease Control and Prevention, Division of Global HIV and TB, 1600 Clifton Rd, NE, US2-1, Atlanta, GA 30329 USA

**Keywords:** female sex workers, prevention of mother-to-child transmission, HIV, HIV-exposed infants, orphans and vulnerable children

## Abstract

As countries strive to eliminate mother-to-child transmission of HIV, female sex workers (FSW) and their children still face barriers to accessing these essential services. Data on FSW uptake of HIV and reproductive health services before, during, and after pregnancy reveal inadequate service utilization. Stigma encountered by FSW in healthcare settings may contribute to low uptake of HIV testing, antiretroviral therapy (ART), and other prevention of mother-to-child HIV transmission (PMTCT) services. Coordination between community-based FSW and facility-based PMTCT programs can facilitate successful linkage of pregnant FSW to antenatal services to support PMTCT efforts. We offer a way forward to reach 90-90-90 targets for FSW and their families and eliminate mother-to-child transmission of HIV.

Nearly four decades into the HIV epidemic, female sex workers (FSW) remain disproportionately burdened compared with other female populations [1]. In low and middle-income countries, the odds of becoming HIV positive is estimated to be 13.5 times greater for FSW than for other reproductive-aged women [2]. HIV prevalence among FSW varies and in Southern Africa, over half of FSW are living with HIV [3–6].

Although data about the HIV epidemic among FSW are becoming increasingly available, there remains a dearth of information about their access to and uptake of sexual and reproductive health (SRH) and prevention of mother-to-child transmission (PMTCT) services. FSW are at risk for unintended pregnancies due to inconsistent condom use, an increased number of sexual partners, and limited access to family planning services [2, 7–9].

The 2016 World Health Organization (WHO) Consolidated Guidelines on the HIV Prevention, Diagnosis and Treatment and Care for Key Populations (KP) includes SRH services amongst the core services to be provided and suggests that PMTCT programs need to consider equity of access, later presentation, and enhanced postpartum and adherence support, but offer no further service delivery recommendations for pregnant, HIV-positive FSW and their infants [10, 11]. Given the challenges faced by FSW in accessing HIV and SRH services geared toward the general population, we suggest a way forward to support SRH and PMTCT services for FSW.

## Sub-optimal SRH and HIV services for FSW

FSW likely face additional hurdles accessing PMTCT services compared to non-sex workers due to vulnerabilities surrounding sex work, including stigma, limited social support, violence, and poverty [12, 13]. When diagnosed with HIV, FSW are often referred to health facilities catering to the general population and whose staff may not be sensitized to FSW or their unique needs [14].

Fear of stigma may prevent FSW from disclosing their sex work to service providers not trained to work with them [15]. In Mozambique, although 94.8% of FSW felt they were treated like everyone else when seeking healthcare, less than half (45.9%) disclosed to healthcare providers that they sold sex [16].

Qualitative interviews with FSW in four southern African countries revealed that FSW experienced stigma and discrimination when accessing health services leading them to not disclose that they sell sex [15]. Enhanced services for FSW during ANC requires that ANC service providers identify FSW and coordinate efforts with FSW programs to ensure systematic stigma-free and confidential follow-up.

In addition to the stigma due to selling sex or living with HIV, pregnant FSW living with HIV may face multi-layered stigma derived from the combination of these factors (e.g., selling sex while pregnant, being HIV positive and pregnant, being an HIV-positive sex worker). FSW, therefore, may encounter negative attitudes from multiple sources when seeking HIV services—service providers, other sex workers, other women accessing health services, and community members [17–20].

Another vulnerability emerges when pregnant FSW transition from FSW-specific services, that is, services targeting only FSW, to ANC services which target all pregnant women. FSW may become effectively “lost to follow up” if linkage between services is not assured, hampering efforts to monitor their ANC uptake. FSW who are not identified as such at ANC may miss out on differentiated care interventions for HIV prevention and care (e.g., pre-exposure prophylaxis (PrEP), post-exposure prophylaxis (PEP), male and female condoms, lubricants, or more frequent HIV testing, and enhanced support for those on ART). Their infants and children may consequently also miss out on opportunities for services, such as those provided by orphans and vulnerable children (OVC) programs.

## Antenatal care (ANC) uptake and HIV testing

Awareness of HIV status among FSW living with HIV ranges from 39.3% in Papua New Guinea to 64.0% in Zimbabwe [6, 21]. HIV testing during ANC is the indispensable first step in PMTCT and can provide a testing avenue for women who may not otherwise be tested. FSW mothers in Burkina Faso were more likely than FSW non-mothers to have tested for HIV, for instance [22]. In Ghana, 45% of FSW who had previously tested for HIV were tested during pregnancy [23]. Though ANC attendance during last pregnancy ranged from 78.5% among FSW in South Sudan to 94.4% in South Africa, uptake of HIV testing at ANC is more variable, ranging from 58.9% in Côte d’Ivoire to 92.0% in South Sudan [4, 24, 25].

## Linkage to HIV treatment, viral suppression, and HIV-exposed infant care

According to a 2014 global review, approximately 42% of all FSW living with HIV were receiving ART [8]. Among all FSW living with HIV in Kampala, Uganda, 37.8% were on ART and 35.2% were virally suppressed [26]. In Zimbabwe, 43.3% of all FSW living with HIV were on ART and 49.5% were virally suppressed, compared to 66.4% and 58.2% among general population women, respectively [6, 27].

Linkage of HIV-infected mothers to ART is critical not only for the mother’s own health, but also for PMTCT [28, 29]. While there are no data on outcomes of HIV-exposed infants (HEI) of FSW, suboptimal awareness of HIV status, linkage to treatment, and viral suppression among FSW suggest their infants may be at a higher risk of MTCT than other infants.

## Breastfeeding and continued care after delivery

If and how quickly FSW mothers return to sex work post-delivery is unclear [3, 22, 25]. Data from Côte d’Ivoire reveal that 58.3% of sex workers returned to selling sex within 4-6 months post-delivery [25]. Women who test HIV-negative prior to delivery but then resume sex work while breastfeeding are at risk of HIV acquisition and subsequent MTCT.

Like their mothers, children of FSW may interact less with the general health system. Data from South Africa have shown that children of FSW, regardless of the mother’s and their own HIV status, experience lower immunization rates and growth than other children [30].

## A call to action

### Essential first steps for including FSW in a vision for the elimination of mother to child transmission of HIV (EMTCT)

Ensuring that high quality comprehensive PMTCT services, implemented according to national guidelines, are accessible to all FSW is the essential first step for service providers and policy makers interested in achieving the World Health Organization’s EMTCT goals [31]. Best practices for increasing service accessibility for KP at the clinic level include: reorganizing clinic setup to increase confidentiality and support disclosure of risk behaviors, non-standard operating hours, and the provision of child care [20].

FSW should be prioritized for PrEP, given their higher risk of HIV acquisition. WHO guidance on Preventing HIV During Pregnancy and Breastfeeding enables prioritization of pregnant, HIV-negative FSW for PrEP [32]. PrEP can facilitate continued FSW engagement with the healthcare system during pregnancy and beyond. Additionally, FSW-focused programs should strengthen the systematic offer of routine HIV testing, provision of male and female condoms, lubricants, post-exposure prophylaxis (PEP), STI screening and treatment, and family planning as needed to give women control of their fertility and reduce the risk of HIV acquisition. FSW programs could further provide HIV self-tests to pregnant FSW and encourage self-testing at regular intervals during pregnancy and breastfeeding [33]. Infants of FSW living with HIV should be considered high risk and prioritized for enhanced infant postnatal prophylaxis and other key HIV-exposed infant services.

For FSW living with HIV, enhanced HIV services during pregnancy and post-partum periods may include provision of linkage to psychosocial support, ART adherence support for those who need it, testing of all their biological children through an index approach, peer mother programs, orphan and vulnerable children services for their families, active case management or follow up, and multi-month ART dispensing . Such additional support is especially important for FSW as they may be younger and have less social support than other women. As little is known about ART adherence among FSW, they may benefit from being prioritized for point-of-care viral load testing in order to provide rapid interventions for elevated viral loads in order to minimize risk of transmission to partners and infants (if pregnant or breastfeeding), and promote improved clinical outcomes.

### Ongoing learning agenda to inform service provision

Better surveillance data and key population disaggregates for program data are needed to characterize the PMTCT and 90-90-90 cascades for FSW and their infants [34]. More information is needed to characterize the risks, vulnerabilities and other experiences of FSW and their families including concerning mental health, protection, economic insecurity, housing stability, food security, and education and immunization of children.

As FSW and PMTCT programs adopt new strategies to reach FSW, they can engage in continuous quality improvement (CQI) to provide immediate feedback from FSW and service providers to inform and adapt services. Furthermore, evaluation of different models of differentiated service delivery for FSW is warranted and could be used to facilitate integration of KP and ANC/PMTCT services. As a richer evidence base is developed from CQI activities, implementation science, program evaluations, and biobehavioral surveys, other innovations may be identified to support the EMTCT agenda among FSW and their children [48].

The lack of data on FSW uptake of PMTCT services should not hinder efforts to improve FSW uptake of essential services. Best practices for providing services to KP can be introduced now in FSW, orphans and vulnerable children, and PMTCT programs to close the gap for pregnant and breastfeeding FSW. Full access to HIV and reproductive health services for FSW, including preventing HIV transmission to their children, is possible. Global stakeholders have the tools available today to ensure the EMTCT agenda is accessible to all families.

## Data Availability

Data sharing is not applicable to this article as no datasets were generated or analysed during the current study.

## Abbreviations

ANC: Antenatal care
ART: Antiretroviral therapy
BBS: Biobehavioral surveys
EMTCT: Elimination of mother-to-child transmission
FSW: Female sex workers
HEI: Hiv-exposed infants
KP: Key populations
OVC: Orphans and vulnerable children
PMTCT: Prevention of mother-to-child transmission
PEP: Post-exposure prophylaxis
PrEP: Preexposure prophylaxis
SRH: Sexual and reproductive health
STI: Sexually transmitted infection
UNAIDS: Joint United Nations Programme on HIV/AIDS
WHO: World Health Organization
